# Decreased olfactory discrimination is associated with impulsivity in healthy volunteers

**DOI:** 10.1038/s41598-018-34056-9

**Published:** 2018-10-22

**Authors:** Aleksandra M. Herman, Hugo Critchley, Theodora Duka

**Affiliations:** 1 Behavioural and Clinical Neuroscience, School of Psychology, University of Sussex, Brighton, BN1 9QH UK; 2 Psychiatry, Department of Neuroscience, Brighton and Sussex Medical School (BSMS), University of Sussex, Brighton, UK; 3 Sackler Centre for Consciousness Science, University of Sussex, Brighton, UK; 4 Sussex Addiction and Intervention Centre, University of Sussex, Brighton, BN1 9QH UK

## Abstract

In clinical populations, olfactory abilities parallel executive function, implicating shared neuroanatomical substrates within the ventral prefrontal cortex. In healthy individuals, the relationship between olfaction and personality traits or certain cognitive and behavioural characteristics remains unexplored. We therefore tested if olfactory function is associated with trait and behavioural impulsivity in nonclinical individuals. Eighty-three healthy volunteers (50 females) underwent quantitative assessment of olfactory function (odour detection threshold, discrimination, and identification). Each participant was rated for trait impulsivity index using the Barratt Impulsiveness Scale and performed a battery of tasks to assess behavioural impulsivity (Stop Signal Task, SST; Information Sampling Task, IST; Delay Discounting). Lower odour discrimination predicted high ratings in non-planning impulsivity (Barratt Non-Planning impulsivity subscale); both, lower odour discrimination and detection threshold predicted low inhibitory control (SST; increased motor impulsivity). These findings extend clinical observations to support the hypothesis that deficits in olfactory ability are linked to impulsive tendencies within the healthy population. In particular, the relationship between olfactory abilities and behavioural inhibitory control (in the SST) reinforces evidence for functional overlap between neural networks involved in both processes. These findings may usefully inform the stratification of people at risk of impulse-control-related problems and support planning early clinical interventions.

## Introduction

Olfactory impairment occurs across neurological and psychiatric disorders, preceding cognitive decline^1–7^. More generally, the assessment of olfactory ability is suggested to offer practical utility as an early marker of a disease progression in neuropsychiatric conditions^8,9^. Importantly, within the brain, olfactory processing shares neurotransmitter systems and neural circuitry with specific cognitive functions. Olfactory information, from receptors in the nasal epithelium, is integrated within the (neurochemically-rich) olfactory bulb, passed directly to medial temporal lobe primary olfactory regions (i.e. piriform cortex, amygdala, and entorhinal cortex) necessary for odour detection. These regions then project directly to secondary olfactory cortex, within the ventral (orbital) prefrontal cortex, which underpins odour discrimination and identification^10^. In the hierarchy of olfactory processing, the ‘peripheral’ ability to detect odours involves olfactory receptors, the olfactory bulb and primary olfactory regions. The ‘higher-order’ ability to discriminate between, identify, and memorise odours, involves the orbitofrontal cortex (OFC)^11^. OFC lesions result in impaired odour discrimination in the presence of intact odour detection^12^. Human neuroimaging studies also show that OFC activation during passive olfactory stimulation^10^ will predict the hedonic experience and motivational impact of odour perception^13^.

The neuroanatomical overlap between the neural substrates for olfactory processing and executive functioning, suggests that olfactory ability can provide an indirect means of assessing the functional integrity of the frontal lobes^11^. More specifically, olfactory identification testing could be used to index OFC functions, including the regulation and inhibition of behaviour^14^.

Clinical studies in patients with obsessive compulsive disorder^15^ and schizophrenia^16^ link olfactory function to the capacity for response inhibition (motor impulsivity). Individuals with post-traumatic stress disorder also show olfactory deficits that predict self-reported levels of impulsivity^17^. Importantly, conditions characterised by increased impulsivity are associated with an atypical sense of smell: Patients with attention-deficit hyperactivity disorder (a neurodevelopmental condition related to inattention, hyperactivity, and impulsive behaviour) show increased odour sensitivity^18^ but impaired odour identification ability^19,20^. People with substance abuse disorders, in whom impulse control is impaired^21–25^, also show olfactory deficits. For example, recently detoxified alcoholics are impaired at odour identification, yet have intact odour detection^26–28^. The olfactory deficits are especially well reported in affective disorders, particularly depression^29,30^. Moreover, elevated impulsivity is also increasingly recognised in depression^31–33^, opening a possibility for a relationship between olfactory abilities and impulsivity in affective disorders as well.

Studies in non-clinical individuals show an association between deficits in odour identification and impaired cognitive abilities, including memory, language, and executive functions^34–37^. For instance, participants with hyposmia performed worse on the Wisconsin Card Sorting Test (a measure of cognitive flexibility) and on the Iowa Gambling Task (IGT; a measure of risky decision making) than normosmic participants^37^. Poorer olfactory discrimination ability is also associated with greater risk-taking and increased sensitivity to immediate rewards on the IGT^38^.

Taken together, these findings, in particular from clinical populations, suggest that tests probing olfactory abilities can provide information about executive functioning and behavioural control and may inform clinical practice as a diagnostic criterion^9,39^ or as a tool for monitoring the treatment^19,40^. Studies with non-clinical populations examining olfactory function and its relationship to cognitive and behavioural functions and in particular to impulsive behaviour appear to be limited. Thus, in the current study, we aimed to characterize the link between olfaction and cognition with a special focus on trait and behavioural impulsivity, in a non-clinical sample of young individuals.

Based on the studies examining the relationship between cognitive functions and olfactory abilities^15–17,37^ and on neuroimaging studies demonstrating commonalities in regional activation patterns between cognitive functions and olfactory abilities (particularly OFC), we hypothesised that deficits in olfactory function would relate to decreased behavioural control of action (increased motor impulsivity on the Stop Signal Task). Regarding trait impulsivity we predicted that Non-planning subscale of the Barratt Impulsiveness Scale might be particularly related to deficits in olfactory abilities. Volumetric studies show that non-planning impulsivity, which assesses lack of future orientation and planning^41^, is associated with grey matter volumes in the OFC^42–44^. In addition, in the present study we explored the relationship between olfactory function and two additional facets of impulsivity: Temporal impulsivity (inability to discount immediate rewards for delayed larger rewards) and reflection impulsivity (reaching decisions with inadequate information).

## Results

Three participants did not undergo olfactory testing due to a common cold (coryza). One male volunteer was (atypically) very inattentive throughout the session and failed to comply with the experimenter’s instructions on three of the tasks. Therefore, his data were excluded from the analyses completely. The final sample consisted of 79 participants (49 females). Additionally, nine participants were excluded from SST analyses as they failed to follow task instructions (i.e. they were slowing down responses when waiting for the stop signal to occur, which resulted in go accuracy below 90% and stop correct rate above 60%). One participant was excluded from the IST FW condition and 2 from the IST RC condition, as they were not sampling information but continuously guessing. Two participants showed extremely low consistency (<75%) on the MCQ and were excluded from that task. For descriptive statistics see Table 1.

**Table 1:** Descriptive statistics of demographic characteristics, trait impulsivity ratings and performance scores in the impulsivity tasks.

	Variable	N Valid	Mean	SD	SE
	Age	79	22.1	3.4	0.4
Alcohol use	Alcohol Units per week	79	12.35	12.21	1.37
	AUQ	79	17.49	16.57	1.87
	Binge Score	79	30.13	25.64	2.88
Smoking	No cigarettes a day	79	0.6	1.7	0.2
Trait impulsivity: BIS	Attention	79	17.56	3.28	0.37
	Motor	79	22.94	3.80	0.43
	Non-Planning	79	23.41	4.52	0.51
	BIS Total	79	63.92	9.12	1.03
SST	SSRT	70	251.73	33.28	3.63
IST	FW P(correct)	78	0.82	0.10	0.01
	RC P(correct)	77	0.73	0.07	0.01
MCQ	log k	77	−2.20	0.62	0.07
Sniffin’ Sticks	Odour detection (sensitivity)	79	8.41	2.04	0.23
	Odour discrimination	79	12.39	2.39	0.27
	Odour identification	79	12.70	1.96	0.22
	Smell Total	79	33.51	4.51	0.51

**Figure 1:**
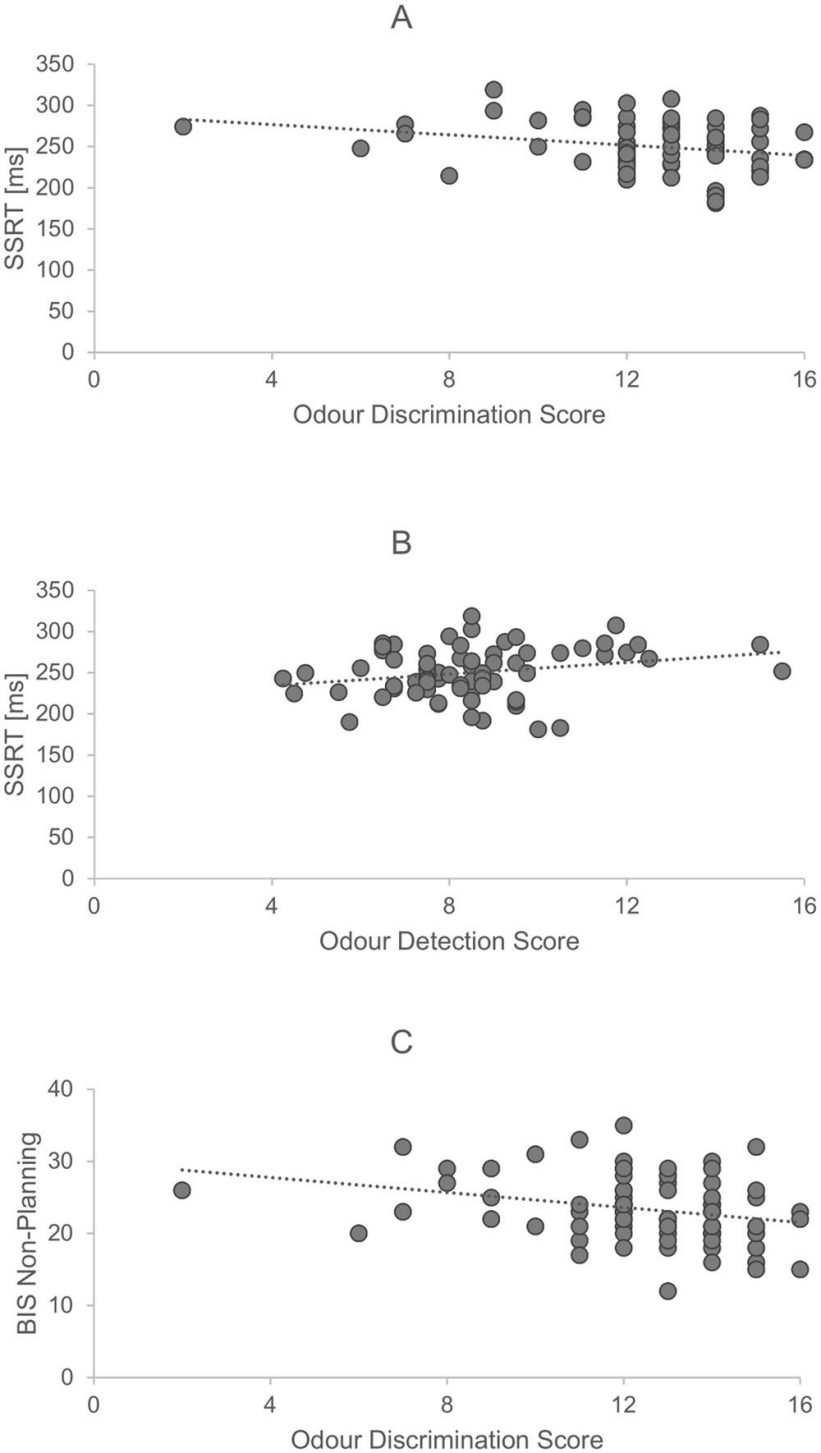
Relationship between olfactory abilities and motor (**A**,**B**) and Non-Planning (**C**) impulsivities. Higher olfactory scores indicate better olfactory abilities.

Regression analysis revealed that only two models, with SSRT and BIS Non-Planning as dependent variables, provided a good fit of the data (Table 2). Odour sensitivity (detection threshold) and odour discrimination were statistically significant predictors of inhibitory control (SSRT; see Table 3, Fig. 1A,B, see also Supplementary Fig. 1), indicating that higher odour sensitivity and lower discrimination predicted poorer pre-potent response inhibition (higher SSRT). Inferior odour discrimination and higher number of cigarettes smoked a day significantly predicted self-reported BIS Non-Planning impulsivity (Table 3, Fig. 1C, see also Supplementary Fig. 1). Exploratory correlations to examine whether the relationship between olfaction and Non-Planning impulsivity might be mediated by the smoking status, revealed that was neither significant correlation between number of cigarettes per day and olfactory discrimination score (*r*(79) = 0.11, *p* = 0.336), nor other olfaction measures (*r*’s ≤ 0.06, *p* ≥ 0.594).The regression models for remaining impulsivity measures were insignificant (*F*’s ≤ 1.37, *p*’s ≥ 0.230, R^2^’s ≤ 0.12).

**Table 2:** Regression models’ summary for analyses of interest.

Model		Sum of Squares	df	Mean Square	*F*	*p*	R	R²
SSRT	Regression	12751.7	7	1821.67	2.22	**0.045**	0.447	0.200
	Residual	50983.87	62	822.32				
	Total	63735.57	69					
BIS Non-Planning	Regression	285.22	7	40.75	2.21	**0.043**	0.423	0.179
	Residual	1309.82	71	18.45				
	Total	1595.04	78

**Table 3:** Predictive capacity of olfactory functions on impulsivity measures: beta-coefficients in linear regressions of interest.

Model		Unstandardized	SE	Standardized	*t*	*p*	Collinearity Statistics	
							Tolerance	VIF
SSRT	(Intercept)	298.99	36.98		8.09	<0.001		
	Threshold	4.50	1.78	0.31	2.53	**0.014**	0.85	1.18
	Discrimination	−3.66	1.61	−0.29	−2.28	**0.026**	0.78	1.29
	Alcohol units a week	0.41	0.35	0.15	1.16	0.249	0.74	1.35
	Gender	−8.43	8.02	−0.13	−1.05	0.297	0.80	1.26
	Cigarettes	−2.15	2.08	−0.13	−1.03	0.306	0.86	1.16
	Age	−1.20	1.26	−0.12	−0.95	0.346	0.80	1.25
	Identification	−0.56	1.99	−0.04	−0.28	0.780	0.79	1.27
BIS Non-Planning	(Intercept)	27.83	5.31		5.24	<0.001		
	Discrimination	−0.58	0.23	−0.31	−2.57	**0.012**	0.79	1.26
	Cigarettes	0.74	0.30	0.28	2.44	**0.017**	0.89	1.13
	Threshold	−0.29	0.26	−0.13	−1.14	0.257	0.87	1.15
	Age	0.11	0.16	0.08	0.69	0.495	0.81	1.24
	Identification	0.18	0.28	0.08	0.64	0.525	0.80	1.25
	Gender	0.13	1.12	0.01	0.11	0.911	0.80	1.26
	Alcohol units a week	0.00	0.05	0.00	0.01	0.993	0.72	1.38

## Discussion

Our study investigated whether higher-order olfactory abilities, namely odour discrimination, can predict cognitive functioning, particularly impulsive behaviour. Taken together, the findings of this study confirm our hypothesis that olfactory functioning relates to both subjective (trait) impulsivity and objective measures of behavioural impulsivity. Therefore, our findings extend earlier demonstrations of the relationship between human olfactory capacity and executive functioning, specifically disadvantageous decision-making and cognitive flexibility^37^. Here, we further characterise the utility of olfaction as a marker for behavioural control function by showing that olfactory discrimination ability predicts the capacity to inhibit pre-potent motor responses (motor impulsivity) and is associated with lower subjective ratings of trait impulsivity in particular ratings reflecting the ability to plan (BIS Non-Planning impulsivity levels). Equally importantly, olfactory abilities were not related to other impulsivity measures.

Our finding that odour discrimination is a significant predictor of low self-reported non-planning impulsivity extends the relationship between olfactory dysfunction and trait impulsivity established in clinical populations^17^. One possible mechanism might be the common brain structures involved. It has been previously shown that the volume of right OFC negatively correlates with BIS non-planning impulsivity^42–44^ and is reduced in hyposmic individuals^45^.

Previous literature has associated olfactory function with inhibitory control; however, this work centres almost exclusively on clinical populations^15,16^. We provide important new evidence within a non-clinical population that broadens and reinforces this association. Moreover, our findings occur in the context of increasing understanding from neuroimaging studies of the neural mechanisms underlying impulsivity. For example, the same SST paradigm used in the current study is known to engage dorsolateral PFC, the inferior frontal gyrus, pre-supplementary motor area, basal ganglia, and insular cortex in the inhibition of pre-potent motor responses^46^.

Importantly, lesion and neuroimaging studies link odour discrimination to the functional integrity of a human brain network encompassing cerebellum, thalamus, caudate, insula, OFC and inferior frontal gyrus^12,47,48^. These latter three regions also underpin the control of motor impulsivity, likely through complementary neurocomputational processes: The inferior frontal gyrus is sensitive to stimulus salience and violations of expectation, responding to the key stimuli in both tasks (stop cues in SST, or ‘the odd one out’ in odour discrimination)^49^.

In addition, our results indicate that higher odour sensitivity (lower detection threshold) is a predictor of higher SSRT, a marker of poorer response inhibition. Thus, in contrast to odour discrimination performance, better odour detection threshold seems to be related to higher motor impulsivity. Olfactory sensitivity is not associated with function of the prefrontal cortex, but instead peripheral olfactory areas. Importantly our findings are in line with the literature on ADHD. Unmedicated patients with ADHD show increased odour detection sesnitivity compared to healthy controls^19,50^ together with hightened motor impulsivity levels, evidenced by poor performance on the SST^51,52^. Interestingly, medication with methylphenidate, known to influence dopaminergic transmission, normalizes odour sensitivity^19^ and simultaneously, improves response inhibition in children with ADHD^53^.

The olfactory bulb is rich in dopaminergic interneurons which inhibit presynaptic olfactory receptos via action on the D2 receptors. D2 receptor agonist quinpirole causes a decrease in odour detection performance, while pretretment with spiperone, D2 receptor antagonist, eliminates those results^54^. It is possible, therefore, that increased odour sensitivity derives from a decrease in dopaminergic activity in the olfactory bulb, and that increase in dopaminergic synaptic availability, mitigates this effect (causes a decrease in detection sensitivity). Dopaminergic system is also implicated in motor impulsivity^55–57^. Together, this evidence indirectly suggests that the relationship between enhanced odour sensitivity and increased motor impulsivity may be mediated by dopaminergic (dys)functioning, which underlies both processes.

Together, our results are consistent with the proposal that odour discrimination has a specific value in predicting the inhibitory control capacity of an individual through indexing the functional integrity of shared neural substrates. Moreover, the findings also suggest a relationship between increased olfactory sensitivity (decreased detection threshold) and poorer response inhibition. Diminished inhibitory control is frequently associated with maladaptive eating behaviour^58,59^, which could also relate more directly to olfactory dysfunction. Thus, the assessment of olfactory function has broad importance in the clinical setting.

Olfactory functioning was not predictive of the ability to delay gratification (temporal impulsivity) or reflection impulsivity. Such findings further suggest that impulsivity is a heterogeneous concept with separate mechanisms^60,61^ and distinct underlying neural networks.

Noteworthy, in our study with normative sample, we found an association between impulsivity measures and odour discrimination but not odour identification abilities. This is in contrast to some previous studies in clinical populations which pointed to the importance of odour identification^15–17^. Possibly, odour identification plays a greater role as a marker of impulsive tendencies in clinical populations, while odour discrimination may be more significant in normative samples. It is important to note, however, that odour discrimination abilities in those aforementioned studies was not assessed at all, suggesting a need for more comprehensive olfactory assessment in clinical samples.

The current study carries several strengths, including a relatively large sample of males and females tested. As our participants were screened for medical history, they were healthy, not suffering from any mental or neurological disorders and not currently taking any medication (apart from hormonal contraceptive pills). Thus, our study shows that olfactory ability is a predictor of cognitive functioning, particularly motor inhibitory control, in a normative population of healthy adults. Most of the previous studies looking at the relationship between olfaction and cognition have tested clinical populations^15–17,26,27,62^ else focus predominantly on memory^35^.

However, there are some limitations to our research. Our sample was selected from the population of university faculty and students. As a result, only young adults (up to 35 years old) were tested, all of whom were high functioning individuals. Additionally, our study did not involve collecting neuroimaging data to confirm the overlap between brain regions involved in the performance of the tasks and olfactory abilities. Therefore, we depended on previously published data for inference about the underlying neuronal circuitry, although the SST used in this study were the same as used in previous neuroimaging studies^46^. Future studies may usefully employ neuroimaging to confirm directly the sharing of neural substrates by olfactory and executive functions. Additionally, our regression models explained only a small proportion of the variance in the dependent variables. This may be due to individuals differences not explored in the current study, such as socioeconomic status or general heath aspects. Finally, only one odorant was used to assess odour detection threshold. Using various odorants with distinct odour qualities (e.g. characteristic unpleasant odours) might be advantageous^63^.

In conclusion, the relationship was established in a group of healthy young adults free of any medications, which makes olfactory abilities an especially good measure of impulsivity in general population and not only in clinical populations. Specifically, our data indicate that good odour discrimination is an especially good predictor of low impulsivity, particularly trait non-planning impulsiveness and pre-potent response inhibition (motor impulsivity), and that odour detection threshold might be an additional predictor of high motor impulsivity.

## Materials and Methods

### Participants

Eighty-three volunteers (50 females) from staff and students of the University of Sussex took part in the study, providing written informed consent. Inclusion criteria were: age 18–35 yrs, fluency in English, no current diagnosis of any mental or neurological disorders, no respiratory allergies, anosmia or cold (coryza), and not taking any medication (other than hormonal contraception). Participants were asked not to drink any caffeine-containing products before testing and to refrain from smoking and eating at least 90 minutes before assessment of olfactory function. The study was approved by the Sciences & Technology Cross-Schools Research Ethics Committee. All the procedures were carried out in accordance with the Ethics Committee guidelines and regulations. Participants were compensated for their time.

### Questionnaires

Each participant completed a battery of questionnaires to assess current mood state, alcohol use, and impulsivity. These data were collected as part of a larger project (see Procedures for details); here we focus on the measurements of cognition, alcohol use, trait impulsivity and olfaction. The Nuffield Hospitals Medical History Questionnaire was used to record demographic details, past and present health status, use of medications and recreational drugs, and an estimate of a number of cigarettes smoked per day. The Barratt Impulsiveness Scale (BIS-11)^41^, a widely used questionnaire in impulsivity research, measured three subtypes of trait impulsivity, namely attentional, motor and non-planning impulsiveness. For the purpose of this study only planning impulsivity subscale will be examined.

### Behavioural tasks

Since impulsivity is a heterogeneous concept^60,61^, a battery of behavioural impulsivity tasks was used to characterise a range of dimensions of the construct.

The Stop Signal Task (SST)^64^ is a measure of motor impulsivity, which assesses the ability to inhibit a pre-potent motor response. Participants respond with button presses to the direction of a green arrow (Go signal) displayed on a computer screen yet are required to withhold this response whenever the arrow changes colour to red (a Stop Signal, occurring on 25% of trials). Participants complete 160 trials.

The Information Sampling Task (IST)^65^ is a measure of reflection impulsivity. On each trial, a matrix of 5 × 5 grey squares was presented on a computer screen. The participant selected a square by clicking with the mouse over the square, to reveal one of two colours (e.g. red and blue) until they were confident which of the two colours was in the majority of the squares. There were two conditions of the task:

(i) Fixed win condition (FW): the participant won 100 points if they made the right decision (regardless of how many boxes they have opened); otherwise, they lost 100 points. The participant completed 10 experimental trials.
(ii) IST reward conflict (RC): for every box opened, the participant lost 10 points from a bank of 250. If the participant chose correctly they won the remaining points from the bank; otherwise, they lost 100 points. Each participant completed 10 experimental trials.

The Monetary Choice Questionnaire (MCQ)^66^ is a measure of temporal impulsivity. The participant was presented with 27 hypothetical choices between small, immediate rewards (SIR) and larger delayed rewards (LDR), for example, “would you prefer £54 today or £55 in 117 days?”. The discounting parameter (k) was calculated for each participant, using the formula: k = ((LDR-SIR)-1)/delay.

The dependent variables for each of the tasks were as follows: On the SST, high Stop Signal Reaction Times (SSRT) indicated high impulsivity; for each condition of the IST (FW and RC), the dependent variable was P(correct) where low values indicated high impulsivity; on the MCQ, a higher mean (log transformed) k value indicated high impulsivity.

### Olfactory testing

Following the cognitive tasks, each participant’s olfactory ability was assessed with the ‘Sniffin’ Sticks’ test (Burghart GmBh, Wedel, Germany). Odorants were presented in felt-tip pens, which instead of a dye contain a tampon with liquid odorants dissolved in propylene glycol. For the odour presentation, the cap was removed, and the stick was held for 2–3 s, approximately 2 cm in front of the participant’s nose.

Three olfactory tests were performed, always in the same order: odour detection threshold, odour discrimination, and odour identification.

Odour detection threshold was assessed using n-butanol according to a single-staircase procedure across the concentration ranges. The participant was presented with three sticks in a randomised order, with two containing the solvent and the third containing a sample concentration the odorant. On each trial, the participant had to identify the odour-containing pen. Presentation of the triplets occurred every 20–30 s until the participant had correctly identified the odorant in two successive trials, which triggered a reversal of the staircase. The procedure lasted until seven reversal points were established. The mean of the last four staircase reversal points was used as the threshold estimate. The scoring ranged from 1 (the lowest odour sensitivity, i.e. the highest detection threshold) to 16 (the highest odour sensitivity, i.e. the lowest detection threshold).

Odour discrimination ability was assessed via three alternative forced-choice procedure. Triplets of pens were presented in a randomised order with two containing the same odorant and one a different one. The participant had to identify which of the pens smelled differently. Sixteen triplets were presented every 20–30 s.

Odour identification was assessed using 16 common odours. Participants had to choose a description matching an odour out of four possibilities. The interval between odour presentations was again 20–30 s.

During threshold and discrimination testing participants were blindfolded with a sleep mask to avoid visual detection of the targets. Each task was scored out of 16, with high scores indicating better olfactory functioning. The scores from each of the tasks together form a “Total score” with a maximum of 48 points.

### Procedure

Upon arrival to the laboratory, the participant was informed about the procedures, signed a consent form, and completed the medical history questionnaire. Next, the participant completed the battery of impulsivity tasks in the randomised order. The study was a part of a larger project testing the relationship between mood state and impulsivity. As part of this larger project all participants underwent a neutral mood induction during a baseline session, preceding the day at which participants received the mood manipulation. All participants viewed neutral images from IAPS database^67^ while listening to music for the duration of five minutes (The Planets, Op. 32: VII. Neptune, the Mystic by Gustav Holst) as a means of mood state normalisation and were asked to provide mood ratings. Data from these measurements are used as baseline measurements for the larger mood induction project (paper in preparation). These procedures were not relevant to the current study; therefore, the results will be described elsewhere. Following the completion of the cognitive tasks, olfactory function was assessed with the Sniffin’ Sticks. The participant was not allowed to smoke, eat, or drink anything but water throughout the session.

### Statistical analysis

Regression analyses evaluated the contribution of olfactory abilities as predictors of distinct aspects of impulsive behaviour. Specifically, we computed a series of multiple regressions with demographical information (sex, age, number of alcohol units consumed a week, number of cigarettes smoked a day) and olfactory scores (odour detection, discrimination and identification scores) as regressors to evaluate predictors for the dependent variables derived from the tasks and from the impulsivity scale. To overcome the problem of multicollinearity between the olfactory scores, Smell Total score was not included into the models.

## Electronic supplementary material

Supplementary Figure 1

## Data Availability

The datasets generated during and/or analysed during the current study are available in the University of Sussex repository, 10.25377/sussex.7172519.

## References

[1] Seidman LJ (1992). Neuropsychological Olfactory probes of fronto-limbic in schizophrenia and Wisconsin system dysfunction Card Sorting Performance. Schizophr. Res..

[2] Moberg PJ (1997). Olfactory identification deficits in schizophrenia: Correlation with duration of illness. Am. J. Psychiatry.

[3] Corcoran C (2005). Olfactory deficits, cognition and negative symptoms in early onset psychosis. Schizophr. Res..

[4] Turetsky BI, Hahn CG, Borgmann-Winter K, Moberg PJ (2009). Scents and nonsense: Olfactory dysfunction in schizophrenia. Schizophr. Bull..

[5] Cohen AS, Brown LA, Auster TL (2012). Olfaction, ‘olfiction,’ and the schizophrenia-spectrum: An updated meta-analysis on identification and acuity. Schizophr. Res..

[6] Rahayel S, Frasnelli J, Joubert S (2012). The effect of Alzheimer’s disease and Parkinson’s disease on olfaction: A meta-analysis. Behav. Brain Res..

[7] Suzuki Y, Critchley HD, Rowe A, Howlin P, Murphy DGM (2003). Impaired olfactory identification in Asperger’s syndrome. J. Neuropsychiatry Clin. Neurosci..

[8] Devanand DP (2000). Olfactory deficits in patients with mild cognitive impairment predict Alzheimer’s disease at follow-up. Am. J. Psychiatry.

[9] Atanasova B (2008). Olfaction: A potential cognitive marker of psychiatric disorders. Neurosci. Biobehav. Rev..

[10] Gottfried JA, Zald DH (2005). On the scent of human olfactory orbitofrontal cortex: meta-analysis and comparison to non-human primates. Brain Res. Brain Res. Rev..

[11] Martzke J, Kopala L, Good K (1997). Olfactory dysfunction in neuropsychiatric disorders: review and methodological considerations. Biol. Psychiatry.

[12] Potter H, Butters N (1980). An assessment of olfactory deficits in patients with damage to prefrontal cortex. Neuropsychologia.

[13] Anderson AK (2003). Dissociated neural representations of intensity and valence in human olfaction. Nat. Neurosci..

[14] Lubman, D. I., Yucel, M. & Brewer, W. J. *In Olfaction and the Brain*. (Brewer, W. J., Castle, D. & Pantelis, C.) 119–132 (Cambridge University Press, 10.1017/CBO9780511543623 (2006).

[15] Bersani G, Quartini A, Ratti F, Pagliuca G, Gallo A (2013). Olfactory identification deficits and associated response inhibition in obsessive-compulsive disorder: On the scent of the orbitofronto-striatal model. Psychiatry Res..

[16] Mauro C.J., Angelo D.D., Hoptman M.J. (2008). OLFACTORY IDENTIFICATION, IMPULSIVITY, AND AGGRESSION IN SCHIZOPHRENIA. Schizophrenia Research.

[17] Dileo JF, Brewer WJ, Hopwood M, Anderson V, Creamer M (2008). Olfactory identification dysfunction, aggression and impulsivity in war veterans with post-traumatic stress disorder. Psychol. Med..

[18] Biederman J, Faraone SV (2005). Attention-deficit hyperactivity disorder. Lancet.

[19] Romanos M (2008). Improved Odor Sensitivity in Attention-Deficit/Hyperactivity Disorder. Biol. Psychiatry.

[20] Ghanizadeh A, Bahrani M, Miri R, Sahraian A (2012). Smell Identification Function in Children with Attention Deficit Hyperactivity Disorder. Psychiatry Investig..

[21] Jentsch JD, Taylor JR (1999). Impulsivity resulting from frontostriatal dysfunction in drug abuse: Implications for the control of behavior by reward-related stimuli. Psychopharmacology (Berl)..

[22] Lubman DI, Yüeel M, Pantelis C (2004). Addiction, a condition of compulsive behaviour? Neuroimaging and neuropsychological evidence of inhibitory dysregulation. Addiction.

[23] Winstanley C (2007). The orbitofrontal cortex, impulsivity, and addiction. Ann N Y Acad Sci..

[24] Perry JL, Carroll ME (2008). The role of impulsive behavior in drug abuse. Psychopharmacology (Berl)..

[25] Schoenbaum G, Shaham Y (2008). The role of orbitofrontal cortex in drug addiction: a review of preclinical studies. Biol Psychiatry..

[26] Ditraglia GM (1991). Assessment of olfactory deficits in detoxified alcoholics. Alcohol.

[27] Shear P, Butters N, Jernigan T (1992). Olfactory loss in alcoholics: correlations with cortical and subcortical MRI indices. Alcohol.

[28] Maurage P (2011). Dissociation between affective and cognitive empathy in alcoholism: A specific deficit for the emotional dimension. Alcohol. Clin. Exp. Res..

[29] Kohli P, Soler ZM, Nguyen SA, Muus JS, Schlosser RJ (2016). The association between olfaction and depression: A systematic review. Chem. Senses.

[30] Taalman H, Wallace C, Milev R (2017). Olfactory functioning and depression: A systematic review. Front. Psychiatry.

[31] Ngo HTT, Street HL, Hulse GK (2011). Is there a relationship between impulsivity and depression in adults? A research synthesis. Curr. Psychiatry Rev..

[32] Swann AC, Steinberg JL, Lijffijt M, Moeller FG (2008). Impulsivity: Differential relationship to depression and mania in bipolar disorder. J. Affect. Disord..

[33] Peluso MAM (2007). Trait impulsivity in patients with mood disorders. J Affect Disord..

[34] Westervelt HJ, Ruffolo JS, Tremont G (2005). Assessing olfaction in the neuropsychological exam: The relationship between odor identification and cognition in older adults. Arch. Clin. Neuropsychol..

[35] Hedner M, Larsson M, Arnold N, Zucco GM, Hummel T (2010). Cognitive factors in odor detection, odor discrimination, and odor identification tasks. J. Clin. Exp. Neuropsychol..

[36] de Guise E (2015). Olfactory and executive dysfunctions following orbito-basal lesions in traumatic brain injury. Brain Inj..

[37] Fagundo AB (2015). Modulation of Higher-Order Olfaction Components on Executive Functions in Humans. PLoS One.

[38] Bettison TM, Mahmut MK, Stevenson RJ (2013). The relationship between psychopathy and olfactory tasks sensitive to orbitofrontal cortex function in a non-criminal student sample. Chemosens. Percept..

[39] Kivity S, Ortega-Hernandez OD, Shoenfeld Y (2009). Olfaction - A window to the mind. Isr. Med. Assoc. J..

[40] Schecklmann M (2011). Effects of methylphenidate on olfaction and frontal and temporal brain oxygenation in children with ADHD. J. Psychiatr. Res..

[41] Patton JH, Stanford MS, Barratt ES (1995). Factor structure of the Barratt impulsiveness scale. J. Clin. Psychol..

[42] Matsuo K (2009). A voxel-based morphometry study of frontal gray matter correlates of impulsivity. Hum Brain Mapp.

[43] Schilling C (2012). Cortical thickness correlates with impulsiveness in healthy adults. Neuroimage.

[44] Cho SS (2013). Morphometric correlation of impulsivity in medial prefrontal cortex. Brain Topogr..

[45] Bitter T (2010). Gray and white matter reduction in hyposmic subjects - A voxel-based morphometry study. Brain Res..

[46] Nikolaou K, Critchley H, Duka T (2013). Alcohol affects neuronal substrates of response inhibition but not of perceptual processing of stimuli signalling a stop response. PLoS One.

[47] Plailly J, Radnovich AJ, Sabri M, Royet JP, Kareken DA (2007). Involvement of the left anterior insula and frontopolar gyrus in odor discrimination. Hum. Brain Mapp..

[48] Savic I, Gulyas B, Larsson M, Roland P (2000). Olfactory functions are mediated by parallel and hierarchical processing. Neuron.

[49] Hampshire A, Chamberlain SR, Monti MM, Duncan J, Owen AM (2010). The role of the right inferior frontal gyrus: inhibition and attentional control. Neuroimage.

[50] Fuermaier Anselm B. M., Hüpen Philippa, De Vries Stefanie M., Müller Morgana, Kok Francien M., Koerts Janneke, Heutink Joost, Tucha Lara, Gerlach Manfred, Tucha Oliver (2017). Perception in attention deficit hyperactivity disorder. ADHD Attention Deficit and Hyperactivity Disorders.

[51] Wolfers T (2016). Quantifying patterns of brain activity: Distinguishing unaffected siblings from participants with ADHD and healthy individuals. NeuroImage Clin..

[52] Senderecka M, Grabowska A, Szewczyk J, Gerc K, Chmylak R (2012). Response inhibition of children with ADHD in the stop-signal task: An event-related potential study. Int. J. Psychophysiol..

[53] DeVito EE (2009). Methylphenidate improves response inhibition but not reflection–impulsivity in children with attention deficit hyperactivity disorder (ADHD). Psychopharmacology (Berl)..

[54] Doty RL, Risser JM (1989). Influence of the D-2 dopamine receptor agonist quinpirole on the odor detection performance of rats before and after spiperone administration. Psychopharmacology (Berl)..

[55] Bari A, Robbins TW (2013). Inhibition and impulsivity: behavioral and neural basis of response control. Prog. Neurobiol..

[56] Bari A, Robbins TW (2013). Noradrenergic versus dopaminergic modulation of impulsivity, attention and monitoring behaviour in rats performing the stop-signal task: Possible relevance to ADHD. Psychopharmacology (Berl)..

[57] Dalley JW, Roiser JP (2012). Dopamine, serotonin and impulsivity. Neuroscience.

[58] Bartholdy S, Dalton B, O’Daly OG, Campbell IC, Schmidt U (2016). A systematic review of the relationship between eating, weight and inhibitory control using the stop signal task. Neurosci. Biobehav. Rev..

[59] Manasse SM (2016). Slowing down and taking a second look: Inhibitory deficits associated with binge eating are not food-specific. Appetite.

[60] Caswell AJ, Bond R, Duka T, Morgan MJ (2015). Further evidence of the heterogeneous nature of impulsivity. Pers. Individ. Dif..

[61] Herman AM, Critchley HD, Duka T (2018). The role of emotions and physiological arousal in modulating impulsive behaviour. Biol. Psychol..

[62] Maurage P, Rombaux P, de Timary P (2013). Olfaction in alcohol-dependence: a neglected yet promising research field. Front. Psychol..

[63] Zufall, F. & Munger, S. D. *Chemosensory Transduction: The Detection of Odors, Tastes, and Other Chemostimuli*. *Chemosens. Transduct. Detect. Odors, Tast. Other Chemostimuli*10.1016/C2014-0-00871-4 (2016).

[64] Gastfriend David R., Garbutt James C., Pettinati Helen M., Forman Robert F. (2007). Reduction in heavy drinking as a treatment outcome in alcohol dependence. Journal of Substance Abuse Treatment.

[65] Clark L, Robbins TW, Ersche KD, Sahakian BJ (2006). Reflection Impulsivity in Current and Former Substance Users. Biol Psychiatry.

[66] Kirby KN, Petry NM, Bickel WK (1999). Heroin addicts have higher discount rates for delayed rewards than non-drug-using controls. J. Exp. Psychol. Gen..

[67] Acharya Naresh, Mithulananthan N. (2007). Locating series FACTS devices for congestion management in deregulated electricity markets. Electric Power Systems Research.

