# Supplementary material for: Decreased olfactory discrimination is associated with impulsivity in healthy volunteers
Source: Sci Rep. 2018 Oct 22;8:15584. doi: 10.1038/s41598-018-34056-9 (PMC6197201; doi:10.1038/s41598-018-34056-9)
Supplement: Supplementary file 1 — Supplementary Figure 1 [file 41598_2018_34056_MOESM1_ESM.pdf]

Decreased olfactory discrimination is associated with impulsivity in healthy volunteers

SUPPLEMANTARY INFORMATION

Aleksandra M. Herman[1*], Hugo Critchley[2,3], Theodora Duka[1,4]

[1]Behavioural and Clinical Neuroscience, School of Psychology, University of Sussex, Brighton, BN1 9QH, UK.

[2]Psychiatry, Department of Neuroscience, Brighton and Sussex Medical School (BSMS), University of Sussex, Brighton, UK

[3]Sackler Centre for Consciousness Science, University of Sussex, Brighton, UK

[4]Sussex Addiction and Intervention Centre, University of Sussex, Brighton, BN1 9QH, UK.

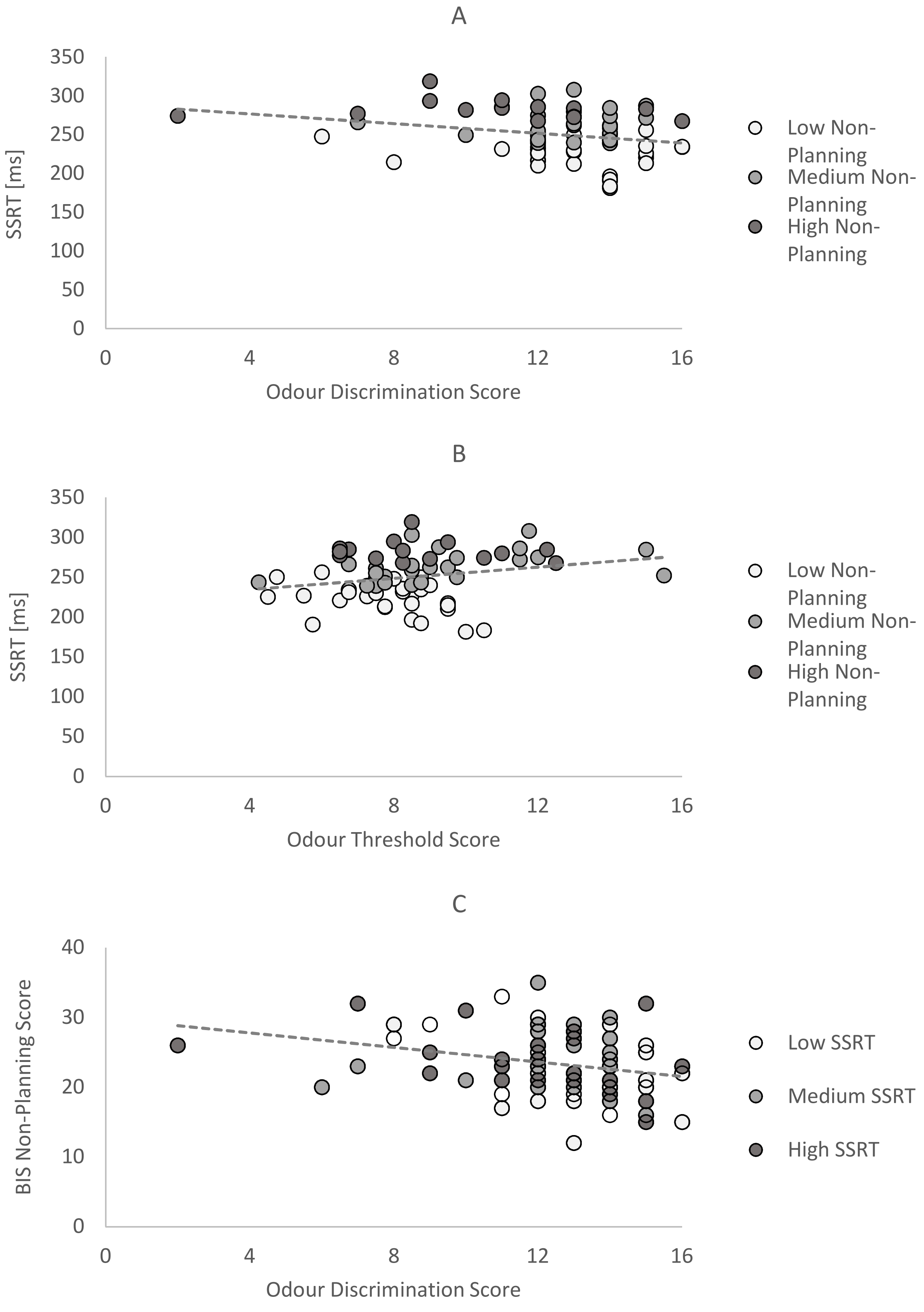

Supplementary Figure 1 Relationship between olfactory abilities and motor (A, B) and Non-Planning (C) impulsivities. Higher olfactory scores indicate better olfactory abilities. Circles in the different shades of grey represent individuals showing low, medium and high impulsivity

levels (separation based on 33rd and 66th percentile split) with regard to BIS non-planning (A and B), and stop signal reaction time (C) for better visualisation of the relationship between different impulsivity measures.
